# CNN Deep Learning with Wavelet Image Fusion of CCD RGB-IR and Depth-Grayscale Sensor Data for Hand Gesture Intention Recognition

**DOI:** 10.3390/s22030803

**Published:** 2022-01-21

**Authors:** Ing-Jr Ding, Nai-Wei Zheng

**Affiliations:** Department of Electrical Engineering, National Formosa University, Huwei, Yunlin 632, Taiwan; 10865110@gm.nfu.edu.tw

**Keywords:** CCD RGB-IR, depth-grayscale, wavelet image fusion, DWT, CNN

## Abstract

Pixel-based images captured by a charge-coupled device (CCD) with infrared (IR) LEDs around the image sensor are the well-known CCD Red–Green–Blue IR (the so-called CCD RGB-IR) data. The CCD RGB-IR data are generally acquired for video surveillance applications. Currently, CCD RGB-IR information has been further used to perform human gesture recognition on surveillance. Gesture recognition, including hand gesture intention recognition, is attracting great attention in the field of deep neural network (DNN) calculations. For further enhancing conventional CCD RGB-IR gesture recognition by DNN, this work proposes a deep learning framework for gesture recognition where a convolution neural network (CNN) incorporated with wavelet image fusion of CCD RGB-IR and additional depth-based depth-grayscale images (captured from depth sensors of the famous Microsoft Kinect device) is constructed for gesture intention recognition. In the proposed CNN with wavelet image fusion, a five-level discrete wavelet transformation (DWT) with three different wavelet decomposition merge strategies, namely, max-min, min-max and mean-mean, is employed; the visual geometry group (VGG)-16 CNN is used for deep learning and recognition of the wavelet fused gesture images. Experiments on the classifications of ten hand gesture intention actions (specified in a scenario of laboratory interactions) show that by additionally incorporating depth-grayscale data into CCD RGB-IR gesture recognition one will be able to further increase the averaged recognition accuracy to 83.88% for the VGG-16 CNN with min-max wavelet image fusion of the CCD RGB-IR and depth-grayscale data, which is obviously superior to the 75.33% of VGG-16 CNN with only CCD RGB-IR.

## 1. Introduction

Human activity recognition [1], which belongs to the categorization of behavior cognition, has been paid much attention in recent years. Human activity information can be represented by two main biometric characteristics: body and hand gesture features. Body gesture-based human activity recognition is generally used in applications of sport instructor experts [2], human–machine interactions by gesture commands [3,4], gesture-based identity recognition [5,6] and rehabilitation tasks\healthcare assistants [7,8,9]. On the other hand, in the field of hand gesture-based human activity recognition, sign language recognition [10,11] and human intention recognition [12] can be practically constructed in real-life applications. Focusing on human activity recognition by hand gesture information, this work proposes a hand gesture-based intention recognition approach by simultaneously considering two different modalities of image data derived from both a charge-coupled device (CCD) and depth cameras.

The popular CCD camera has been successfully used in surveillance applications. To have the capability of night vision (allowing the camera to see in the dark), the CCD camera is generally designed to have numerous infrared (IR) LEDs surrounding the image sensor. With the light emitted by infrared illuminators, such captured image from the CCD camera is well-known as CCD RGB-IR. Although CCD RGB-IR images have a relatively fine image-rendering property in an environment with low illumination (or completely dark), such images are somewhat substandard in the task of human activity recognition. For image recognition, including hand gesture recognition, in this work, the use of sensor fusion by additionally increasing and hybridizing another different image sensing modality of data will have positive effects on recognition accuracy improvements. Studies on IR image-based sensor fusion have been seen in the recent years. Most of these works are aimed at fusion of visible (VIS) and specific IR images [13,14,15,16,17,18,19]. In the study of Hu et al., a synchronized fusion model is proposed for multi-band images where images of far-infrared (FIR), near-infrared (NIR) and VIS are encompassed [13]. An adaptive fusion algorithm for VIS and IR videos is proposed in [14], which is based on entropy and the cumulative distribution of gray levels. In [15], a fusion network model, called a relativistic coupled generative adversarial network (RCGAN), is developed for the IR and VIS image fusion task. Another enhancement of the generative adversarial network on image fusion can also be seen in the work of [16], in which a dual-discriminator conditional generative adversarial network (DDcGAN) is designed to fuse IR and VIS images of different resolutions; in addition, fusion of VIS and IR images by a generative adversarial network is also employed in intelligent ubiquitous electric internet of things (UE-IoT), to detect fault points in a more reliable and accurate manner [17]. In [18], IR and VIS image fusion is used in image-based object tracking. To improve dissatisfactory object tracking in the situation that unreliable VIS images are captured in poor illumination conditions, a visible RGB and IR fusion tracking approach that is based on the fully convolution Siamese networks has been proposed by Zhang et al. [18]. In turn, Hou et al. proposes a framework called VIF-Net, and with VIF-Net, two different types of data from the VIS and IR sensors are used for the feature extraction, fused, and then the feature is reconstructed [19].

Although lots of image fusion approaches have been investigated, most of these studies mainly combine the two advantages of night-visible capability and rich texture information obtained from IR and VIS-RGB, respectively. In a CCD camera-based surveillance scenario with human activity recognition, such fusion of VIS and IR images for further hand gesture activity recognition will perhaps not be feasible. During nighttime of darkness, i.e., an environment with extremely low illumination, it will be difficult to obtain the information from VIS images. In this situation with insufficient light (darkness approaching), only the CCD RGB-IR image can be acquired from the CCD camera. However, human activity recognition using only CCD RGB-IR with the restricted texture details will not have perfect recognition performances. In some studies [20,21,22,23], thermal image-based approaches to use images of infrared thermography (IRT) acquired from the sensing device of the IR thermal imager for analyzing activity gesture information are presented to maintain a satisfactory recognition accuracy even in an adverse environment of low lights. However, a high cost problem of the IR thermal imager will be inevitably encountered, which will be an adverse factor on the market acceptance to such intelligent applications. To tackle this issue, in this paper, depth image information derived from a depth sensor that belongs to the sensor category of time of flight (ToF) is additionally considered to be fused with the CCD RGB-IR data for constructing hand gesture intention recognition with outstanding performance. It is well-known that a depth sensor will not be restricted in performance by the illumination factor. A hand gesture intention recognition system using convolution neural network (CNN) deep learning incorporated with wavelet image fusion of CCD RGB-IR and depth-grayscale data is proposed in this work, which will be detailed in the following sections.

The primary contributions of this work are summarized and listed as follows:

(1) An effective deep learning recognition framework, CNN, incorporated with wavelet image fusion of the dual modalities of sensor data, CCD RGB-IR and depth-grayscale, is proposed for hand gesture intention recognition.
(2) Compared with traditional CNN deep learning and recognition using only a single modality of sensor data (either CCD RGB-IR or depth-grayscale), the presented CNN with wavelet image fusion of both CCD RGB-IR and depth-grayscale has obvious and significant performance impacts on gesture recognition accuracy.
(3) Compared with those studies using fusion of VIS and IR images in CCD camera-based surveillance applications with human activity recognition, gesture recognition using a fusion of CCD RGB-IR and depth-grayscale, as per the presented approach, will be much competitive, especially in adverse conditions such as darkness or low illumination.
(4) Compared with those works by IR thermal image-based approaches for overcoming the problem of gesture recognition in the condition of low lights, the presented approach will be much more advantageous and acceptable given the costs of sensor deployments.

The remainder of this paper is organized as follows. Section 2 provides a primary description of the typical calculation framework of CNN deep learning in a general recognition task. Section 3 details the hand gesture intention recognition using the presented CNN deep learning incorporated with wavelet image fusion of the CCD RGB-IR and depth-grayscale sensing data. Section 4 presents the experiment results where the effectiveness and performance of the proposed CNN with wavelet image fusion of CCD RGB-IR and depth-grayscale are demonstrated, compared with conventional CNN with only CCD RGB-IR alone or depth-grayscale alone. Section 5 is a discussion of related techniques and real-world applications. Finally, Section 6 gives some concluding remarks.

## 2. Typical VGG-16 CNN Deep Learning on Recognition

Convolution neural network-based deep learning has been successfully used in pattern recognition applications, including hand gesture intention recognition in this work. Compared with the traditional artificial neural network (i.e., the so-called ANN) scheme, additional convolution and pooling computation layers are finely incorporated inside the CNN model. Convolution and pooling tasks in CNN are mainly to perform image feature extraction by a series of filters and reduce the data dimension of the extracted feature information, respectively. Each convolution computation is followed by a corresponding pooling process. With such layer-by-layer convolution and pooling calculations on the set of input images, the CNN model can finally achieve the purpose of image characteristics learning in a deep manner.

**Figure 1:**
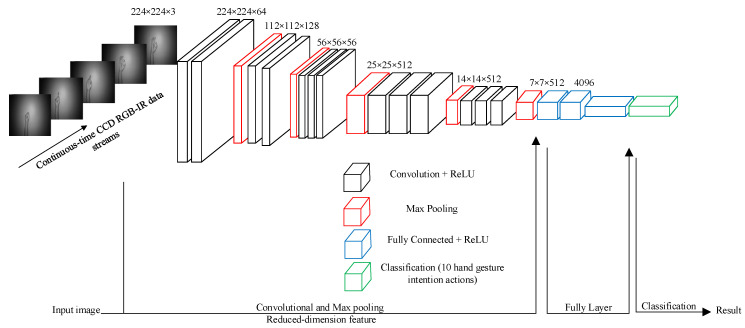
Frameworks of VGG-16 CNN deep learning [24] for the continuous-time hand gesture data stream (10 gesture classification nodes set in the final layer).

In this study of hand gesture intention recognition, the well-known VGG-16 CNN model is adopted [24]. The VGG-16 CNN structure was developed by the visual geometry group (VGG) for constructing 2-dimentional (2-D)-based image recognition system. As can be seen in Figure 1, the calculation framework of VGG-16 CNN contains two main process parts: the first part of convolution and max pooling estimates (13 layers) and the second part of fully connected (FC) classification computations (3 layers). In this study, a set of continuous-time RGB-IR hand gesture images (each image fixed to the size of 224 by 224) that denote different classifications of human intentions was sent to VGG-16 CNN for learning and recognition. It can be noted that in the last layer of VGG-16 CNN (i.e., the final classification layer), the node number in this layer is set to 10 to denote class match values of 10 corresponding hand gesture intention categorizations.

**Figure 2:**
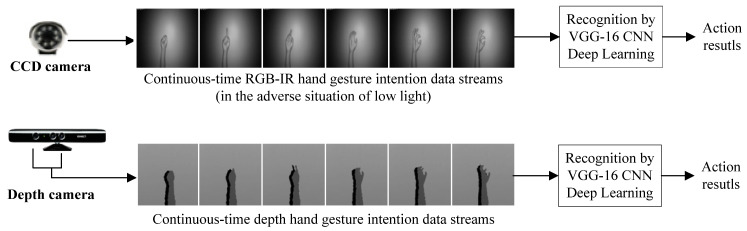
The typical VGG-16 CNN deep learning recognition framework with only one modality channel to process the same type of sensor data (the CCD RGB-IR modality or the depth camera-derived depth-grayscale modality in an environment of low illumination).

Figure 2 depicts a typical one-channel VGG-16 CNN deep learning recognition scheme without consideration of the fusion of different modalities of sensor data. It is clearly seen in Figure 2 that for the CCD camera-derived data stream, the RGB-IR hand gesture data-based VGG-16 CNN can be trained and then built up. Such single-channel VGG-16 CNN deep learning using only the same modality data of RGB-IR will inevitably encounter the problem of dissatisfactory recognition performance in situations where the recognition environment is lowly illuminated (or in darkness). For overcoming this problem and further enhancing such single-channel CCD RGB-IR VGG-16 CNN gesture recognition, a dual-channel VGG-16 CNN deep learning framework by incorporating the wavelet-based image fusion scheme to effectively hybridize two different modalities of sensor data, CCD RGB-IR and depth-grayscale (from a depth camera), is presented, which will be detailed in Section 3. The single-channel VGG-16 CNN deep learning framework of the depth camera-derived depth-grayscale data is also provided in Figure 2 for clearness on recognition performance comparisons.

## 3. Hand Gesture Intention Recognition by Presented VGG-16 CNN Deep Learning Incorporated with Wavelet Fusion of CCD RGB-IR and Depth-Grayscale Sensing Images

**Figure 3:**
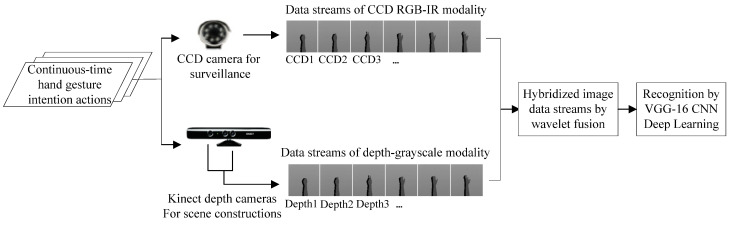
The proposed dual-channel sensor fusion recognition approach using VGG-16 CNN deep learning incorporated with wavelet-based image fusion of CCD RGB-IR and depth-grayscale for hand gesture intention recognition.

**Figure 4:**
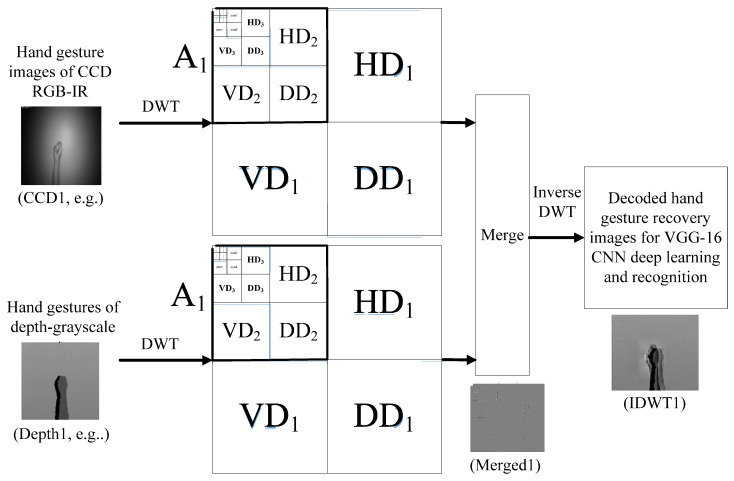
The wavelet transform fused-data stream for VGG-16 CNN deep learning and recognition acquired by DWT decomposition of the input image, DWT filter coefficient merge and inverse DWT to obtain the recovery image.

In this work, wavelet-based image fusion is properly incorporated into the VGG-16 CNN hand gesture intention recognition for obtaining reliable recognition outcomes. For wavelet image fusion, it is mainly composed of three estimate procedures, which are (1) extraction of filter coefficients (i.e., DWT coefficients) of the CCD RGB-IR and depth-grayscale sensor images; (2) merge computations of the derived DWT coefficients of the two different sensor data types; and (3) inverse discrete wavelet transform (IDWT) carried out for the merged image for decoding and getting back a recovery image. The IDWT-decoded recovery image is then sent to the VGG-16 deep learning model for establishment and classification of the training models of the hand gesture intention actions. Figure 3 depicts the presented dual-channel sensor fusion approach to hybridize the CCD RGB-IR and depth-grayscale sensor image data by wavelet fusion for VGG-16 deep learning hand gesture intention action recognition. Figure 4 shows the hybridized action data stream for VGG-16 CNN deep learning and recognition derived from the mentioned three procedures in wavelet image fusion.

### 3.1. Discrete Wavelet Transform of Five Levels for Decompositions of CCD RGB-IR and Depth-Grayscale Hand Gesture Action Data

**Figure 5:**
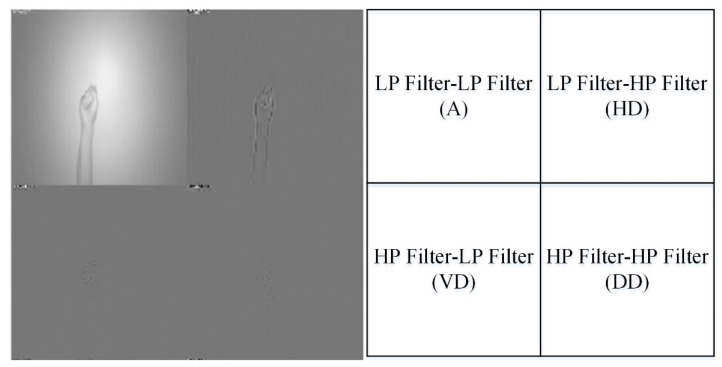
Four separated decomposition components, image data of the approximate (*A*), detailed data of the horizontal (*H*), detailed data of the vertical (*V*) and detailed data of the diagonal (*D*), derived from discrete wavelet transform (one-level DWT decomposition on CCD RGB-IR, for example).

As mentioned, in the wavelet-based image fusion, each of two different sensor modality data of the acquired hand gesture intention action image is first used to derive the corresponding filter coefficients using wavelet transform. The well-known wavelet transformation of the image with two-dimensional (*x*,*y*) pixel raw data is essentially categorized into two-dimensional wavelet transformation, which performs an encoding procedure using the original input image. The discrete wavelet transform will separate the original image to four different independent segments, which are the approximated (*A*) image part, the horizontal detail (*HD*) data part, the vertical detail (*VD*) data and the diagonal detail (*DD*) data. Figure 5 shows an example that one-level DWT decomposition is performed on a hand gesture image with the sensor modality of CCD RGB-IR. It is clearly seen that four decomposed components, *A*, *HD*, *VD* and *DD*, are extracted after the one-level DWT process. Note that the approximated image part will keep most of the original image data, and it is obtained by calculations of low-pass (LP) filter and LP filter (i.e., the *LL* decomposition coefficient); the *HD* segment is derived using the LP filter and the high-pass (HP) filter (i.e., the *LH* decomposition coefficient); the HP filter and the LP filter are employed to determine the VD segment (i.e., the *HL* decomposition coefficient); finally, the *DD* segment can be estimated using two high-pass filters, HP filter-HP filter (i.e., the *HH* decomposition coefficient). These *LL*, *LH*, *HL* and *HH* wavelet decomposition coefficients that are representative of those corresponding regions, *A*, *HD*, *VD* and *DD*, respectively, can be estimated using Equations (1)–(4), as follows.

(1) $$A_{i}:\;LL_{i}\left(x,y\right)=\sum_{n=0}^{K-1}\sum_{m=0}^{K-1}LL_{i-1}\left(m,n\right)\times l\left(2x-m\right)\times l\left(2y-n\right),\;x,y\in Z,\;K=224,\;i=1,2,\ldots ,5.$$

(2) $$VD_{i}:\;HL_{i}\left(x,y\right)=\sum_{n=0}^{K-1}\sum_{m=0}^{K-1}LL_{i-1}\left(m,n\right)\times h\left(2x-m\right)\times l\left(2y-n\right),\;x,y\in Z,K=224,\;i=1,2,\ldots ,5.$$

(3) $$HD_{i}:\;LH_{i}\left(x,y\right)=\sum_{n=0}^{K-1}\sum_{m=0}^{K-1}LL_{i-1}\left(m,n\right)\times l\left(2x-m\right)\times h\left(2y-n\right),\;x,y\in Z,K=224,\;i=1,2,\ldots ,5.$$

(4) $$DD_{i}:\;HH_{i}\left(x,y\right)=\sum_{n=0}^{K-1}\sum_{m=0}^{K-1}LL_{i-1}\left(m,n\right)\times h\left(2x-m\right)\times h\left(2y-n\right),x,y\in Z,\;K=224,i=1,2,\ldots ,5.$$

Note that in Equations (1)–(4), l(2x−m) and l(2y−n) denote low-pass filters, and h(2x−m) and h(2y−n) represent high-pass filters; the index *i* is the level number of iterative DWT decomposition levels. These four terms, $LL_{i}\left(x,y\right)$, $HL_{i}\left(x,y\right)$, $LH_{i}\left(x,y\right)$ and $HH_{i}\left(x,y\right)$, denote the approximated image part, the vertical detail data part, the horizontal detail data part and the diagonal detail data part in the *i*-th level DWT decomposition, respectively. Parameters *x* and *y* are the *x*-axis and *y*-axis positions of a pixel in an input image, respectively. The maximum value of both *x* and *y* is set to K − 1 (K = 224, as shown in Equations (1)–(4); images with the invariable size of 224 by 224 required for specific deep learning of VGG-16 CNN, as introduced in Section 2). In a series of multi-level DWT decomposition calculations, the approximated image part, $LL_{i}\left(x,y\right)$, keeps crucial information about the pixel raw data in the *i*-th decomposition level, by which the DWT decomposition parameter sets of the next level (i.e., *i* + 1) can then be iteratively estimated. It is also noted that the term $LL_{i}\left(x,y\right)$ with *i* set to 0, i.e., $LL_{0}\left(x,y\right)$, denotes the original input image before performing DWT.

**Figure 6:**
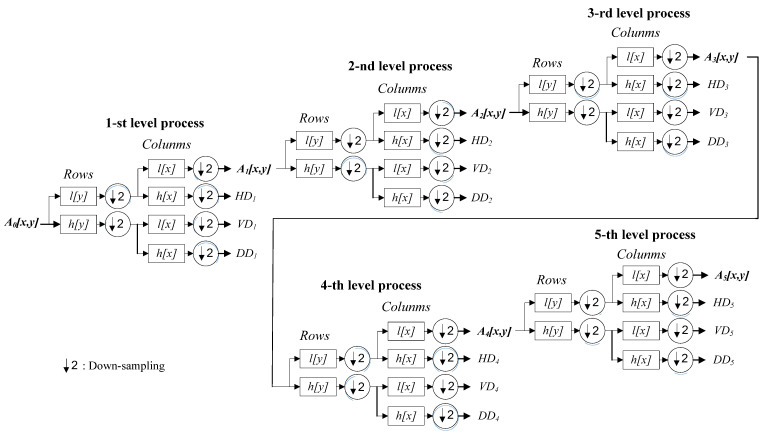
Five-level DWT calculations employed for each of the CCD RGB-IR and depth-grayscale sensor data modalities (flowcharts of the approximate segment in each phase-performed decomposition).

In this study, 5-level DWT, which contains five consecutive phases of data decomposition (i.e., i=5), is adopted to encode each of the CCD RGB-IR and depth-grayscale sensing data modalities (see Figure 6). Compared with only one-level DWT data encoding in Figure 5, the 5-level DWT estimate, as shown in Figure 6, will continually perform five phases of data decomposition calculations. As can be seen in Figure 6, when completing the 1st DWT on the original input image, the approximate data *A*_1_, and three segments of detailed data, *HD*_1_, *VD*_1_ and *DD*_1_, can be extracted. The derived approximate image data *A*_1_ will then be used as the input image of the 2nd level DWT. The approximate data *A*_2_ and other three segments of detailed data, *HD*_2_, *VD*_2_ and *DD*_2_ can be obtained in the 2nd DWT data decomposition. Similar calculations are done in the 3rd, 4th and 5th level DWT data decomposition. Note that, in this work, after performing 5-level DWT estimates on each of the CCD RGB-IR and depth-grayscale modality data, five coefficient parameter sets (i.e., (*A*_1,*CCD*_, *HD*_1,*CCD*_, *VD*_1,*CCD*_, *DD*_1,*CCD*_), (*A*_2,*CCD*_, *HD*_2,*CCD*_, *VD*_2,*CCD*_, *DD*_2,*CCD*_),…, and (*A*_5,*CCD*_, *HD*_5,*CCD*_, *VD*_5,*CCD*_, *DD*_5,*CCD*_)) and another 5 coefficient parameter sets (i.e., (*A*_1,*Depth*_, *HD_1_*_,*Depth*_, *VD*_1,*Depth*_, *DD*_1,*Depth*_), (*A*_2,*Depth*_, *HD*_2,*Depth*_, *VD*_2,*Depth*_, *DD*_2,*Depth*_), …, and (*A*_5,*Depth*_, *HD*_5,*Depth*_, *VD*_5,*Depth*_, *DD*_5,*Depth*_)) can then be used to represent the input sensing images of CCD RGB-IR and depth-grayscale, respectively.

Following DWT data decomposition, a merge computation to hybridize these two different modalities of DWT coefficient parameter sets of CCD RGB-IR and depth-grayscale in each of the five levels will be done. Finally, an inverse DWT (IDWT) estimate will be performed on the merged data (i.e., data decoding), where a recovered image with pixel-based raw data can then be obtained and further used for CNN deep learning and recognition, which will be detailed in Section 3.2.

**Figure 7:**
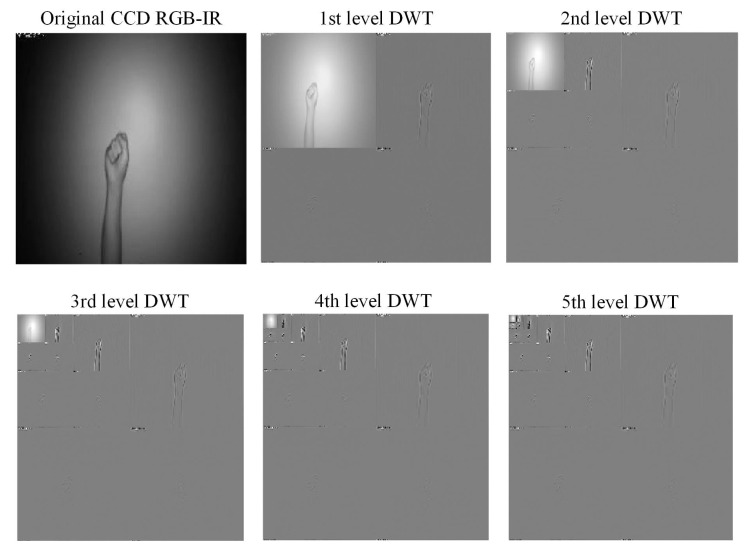
Coefficient derivation process of 5-level DWT decomposition on sensing data of the CCD RGB-IR modality of the hand gesture action.

**Figure 8:**
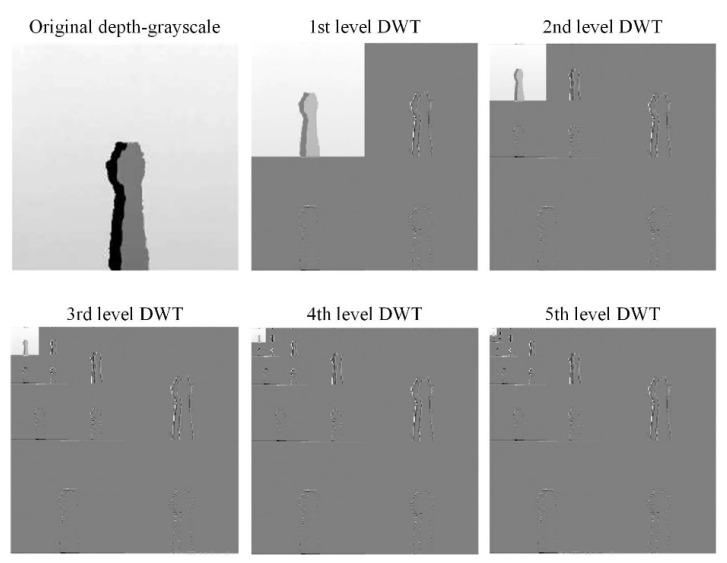
Coefficient derivation process of 5-level DWT decomposition on sensing data of the depth grayscale modality of the hand gesture action.

Figure 7 and Figure 8 are the coefficient derivation process of the 5-level DWT decomposition on two different modalities of hand gesture sensing data, namely, CCD RGB-IR and depth-grayscale, respectively. As shown in Figure 7 and Figure 8, the approximated data region in the overall image will become smaller and smaller with an increasing DWT decomposition level. In contrast, when the iterative number of such a DWT decomposition is increased, there are more and more detailed data contained in the image. Note that in merging the calculations of the two different modalities of wavelet decomposition coefficients, the decomposition coefficient of the two different sensing modalities of the image data with the same DWT decomposition level number will be taken into account, with coefficient hybridizations done on both of the 5th level DWT coefficient of CCD RGB-IR (see Figure 7) and the 5th level DWT coefficient of the depth-grayscale (see Figure 8), for example.

### 3.2. Decomposition Data Merge and IDWT-Decode to Derive Hybridized Data Streams for VGG-16 CNN Deep Learning on Hand Gesture Intention Action Recognition

As mentioned, in wavelet-based image fusion, after finishing the extraction of the DWT decomposition coefficients of each of the CCD RGB-IR and depth-grayscale sensor images, a coefficient merge process will then be followed. In this work, three data fusion strategies, max-min, min-max and mean-mean, to merge the CCD RGB-IR and depth-grayscale DWT decomposition coefficients in each DWT calculation level, were employed. The following details the three general DWT image fusion strategies used in this work. Note that additional merging schemes, such as substitutive wavelet fusion, additive wavelet fusion and weighted model wavelet fusion, also have been employed in image-related applications [25,26].

(1) The data fusion strategy of max-min

In each DWT decomposition level, the merge operation of max-min for hybridizing CCD RGB-IR and depth-grayscale DWT decomposition coefficients is to decide the maximum between the approximate data of CCD RGB-IR and depth-grayscale; in addition, for each of the regions of vertical, horizontal and diagonal detailed data, the merge operation is to estimate the minimum between the CCD RGB-IR and depth-grayscale sensor modality decomposition parameters. Equations (5)–(8) detail the max-min data fusion strategy.

(5) $$\;A_{i,Merged}=max(A_{i,\;CCD},A_{i,Depth}),\;i=1,2,\ldots ,5.$$

(6) $$\;VD_{i,Merged}=min(VD_{i,\;CCD},VD_{i,Depth}),\;i=1,2,\ldots ,5.$$

(7) $$HD_{i,Merged}=min(HD_{i,\;CCD},HD_{i,Depth}),\;i=1,2,\ldots ,5.$$

(8) $$\;DD_{i,Merged}=min(DD_{i,\;CCD},DD_{i,Depth}),\;i=1,2,\ldots ,5.$$

(2) The data fusion strategy of min-max

Compared with the abovementioned max-min fusion operation, the min-max data fusion strategy herein is to make the minimum decision between the CCD RGB-IR and depth-grayscale approximate data and find the maximum between the CCD RGB-IR and depth-grayscale detailed data in each of the five DWT decomposition levels. Equations (9)–(12) show the calculation of the min-max data fusion strategy.

(9) $$\;A_{i,Merged}=min(A_{i,\;CCD},A_{i,Depth}),\;i=1,2,\ldots ,5.$$

(10) $$\;VD_{i,Merged}=max(VD_{i,\;CCD},VD_{i,Depth}),\;i=1,2,\ldots ,5.$$

(11) $$HD_{i,Merged}=max(HD_{i,\;CCD},HD_{i,Depth}),\;i=1,2,\ldots ,5.$$

(12) $$\;DD_{i,Merged}=max(DD_{i,\;CCD},DD_{i,Depth}),\;i=1,2,\ldots ,5.$$

(3) The data fusion strategy of mean-mean

Different to the max-min and min-max merge operations, the mean-mean data fusion strategy simultaneously takes into consideration the CCD RGB-IR and depth-grayscale decomposition information in the data merge computations of each level. The mean-mean data fusion is essentially similar to additive wavelet fusion. The additive fusion strategy is that the approximate information of one modality data is added by that of the other modality data. The detailed data of the two different input modalities are also extracted using the same operation. In this work, mean-mean data fusion is adopted to further derive the averaged information of the accumulative CCD RGB-IR and depth-grayscale approximate and detailed data. Detailed operations of the mean-mean data fusion strategy can be clearly seen in Equations (13)–(16).

(13) $$\;A_{i,Merged}=mean(A_{i,\;CCD},A_{i,Depth}),\;i=1,2,\ldots ,5.$$

(14) $$\;HL_{i,Merged}=mean(HL_{i,\;CCD},HL_{i,Depth}),\;i=1,2,\ldots ,5.$$

(15) $$LH_{i,Merged}=mean(LH_{i,\;CCD},LH_{i,Depth}),\;i=1,2,\ldots ,5.$$

(16) $$\;HH_{i,Merged}=mean(HH_{i,\;CCD},HH_{i,Depth}),\;i=1,2,\ldots ,5.$$

**Figure 9:**
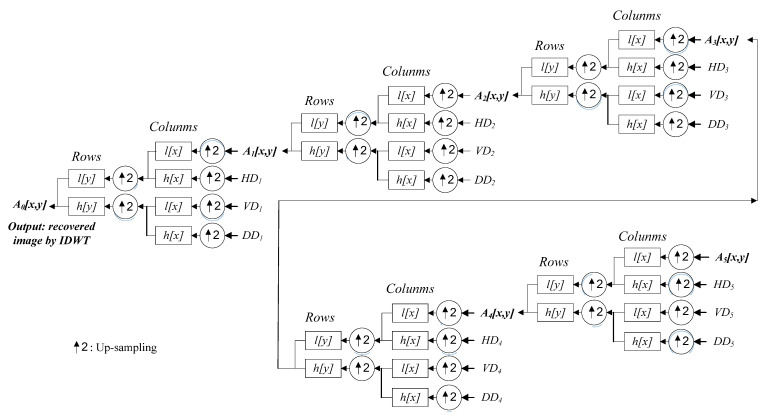
Five-level IDWT calculations employed in this work for finally obtaining the recovery images with pixel information (flowcharts of the IDWT decoding of each of the five levels).

Following the data fusion process, for the merged image is then performed the inverse discrete wavelet transform to make a series of inverse operations of DWT calculations (i.e., decoding of the DWT data). After completing the IDWT computation, the DWT decomposition data-merged image will then be transformed and returned to the recovery image with all pixel raw information. Equation (17) shows the 5-level IDWT decoding process, which is a computation of pixel-based image recovery. Note that, in Equation (17), the recovery image of the corresponding 5-level IDTW calculation can finally be decoded and acquired in the case of i=0, which is the term $LL_{0}\left(x,y\right)$ (i.e., $A_{0}\left(x,y\right)$). The IDWT-image, $LL_{0}\left(x,y\right)$, will then be viewed as the input data of the VGG-16 model to further perform deep learning and recognition of the hand gesture intention actions. It is also noted that the image of $LL_{0}\left(x,y\right)$ reveals the hybridization information of the two original input images of CCD RGB-IR and depth-grayscale. Figure 9 illustrates the 5-level IDWT decoding process for achieving pixel-based image recovery from a series of wavelet decomposition coefficients derived from 5-level DWT, $A_{5}$, *HD*_5_, *VD*_5_, *DD*_5_, *HD*_4_, *VD*_4_, *DD*_4_, *HD*_3_, *VD*_3_, *DD*_3_, *HD*_2_, *VD*_2_, *DD*_2_, *HD*_1_, *VD*_1_ and *DD*_1_.

(17) $$\begin{array}{l} LL_{i}\left(x,y\right)=\;\sum_{m=-\infty }^{\infty }\sum_{n=-\infty }^{\infty }LL_{i+1}\left(m,n\right)\;\times \;l\left(y-2n\right)\;\times \;l(x\;-\\ 2m)+\sum_{m=-\infty }^{\infty }\sum_{n=-\infty }^{\infty }HL_{i+1}\left(m,n\right)\;\times \;l\left(y-2n\right)\;\times \;h\left(x-2m\right)\;+\\ \sum_{m=-\infty }^{\infty }\sum_{n=-\infty }^{\infty }LH_{i+1}\left(m,n\right)\;\times \;h\left(y-2n\right)\;\times \;l\left(x-2m\right)+\\ \sum_{m=-\infty }^{\infty }\sum_{n=-\infty }^{\infty }HH_{i+1}\left(m,n\right)\;\times \;h\left(y-2n\right)\;\times \;h\left(x-2m\right),\;x,y\in Z,\;i=4,3,\ldots ,0. \end{array}$$

**Figure 10:**
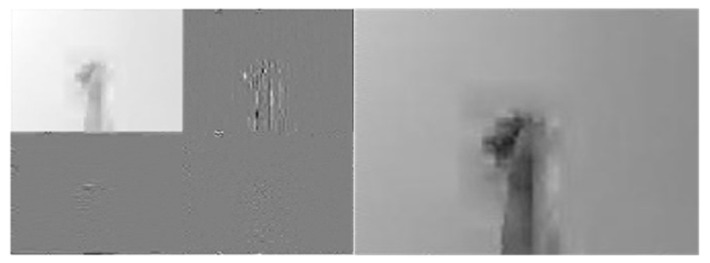
The “max-min” merge strategy performed on two 1st-level DWT coefficients derived from CCD RGB-IR and depth-grayscale hand gesture images (**the left**) and the IDWT-decoded recovery image of the “max-min” merged (**the right**) for VGG-16 CNN deep learning.

**Figure 11:**
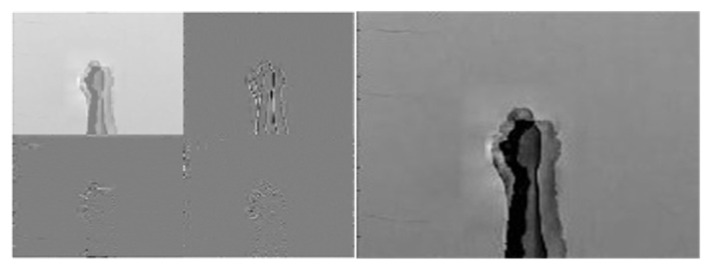
The “min-max” merge strategy performed on two 1st-level DWT coefficients derived from CCD RGB-IR and depth-grayscale hand gesture images (**the left**) and the IDWT-decoded recovery image of the “min-max” merged (**the right**) for VGG-16 CNN deep learning.

**Figure 12:**
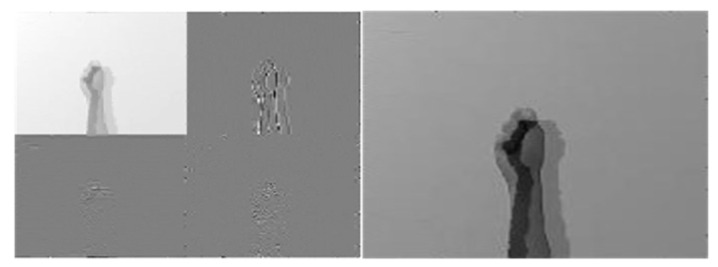
The “mean-mean” merge strategy performed on two 1st level DWT coefficients derived from CCD RGB-IR and depth-grayscale hand gesture images (**the left**) and the IDWT-decoded recovery image of the “mean-mean” merged (**the right**) for VGG-16 CNN deep learning.

Figure 10, Figure 11 and Figure 12 depict wavelet image fusion of CCD RGB-IR and depth-grayscale by the DWT decomposition coefficient fusion strategies of max-min, min-max and mean-mean, respectively. For clearly observing the difference of each fusion strategy on the merged image and its corresponding IDWT-decoded image, only one-level wavelet image fusion information is shown. As shown in Figure 10, the left represents the max-min merged image information, i.e., the set of four segments of $\;A_{1,Merged}$, $\;VD_{1,Merged}$, $HD_{1,Merged}$ and $\;DD_{1,Merged}$, which are derived from the computation of $max(A_{1,\;CCD},A_{1,Depth})$, $min(VD_{1,\;CCD},VD_{1,Depth})$, $min(HD_{1,\;CCD},HD_{1,Depth})$ and $min(DD_{1,\;CCD},DD_{1,Depth})$, respectively (see Equations (5)–(8)). The right of Figure 10 denotes the recovery image from calculations of IDWT on the max-min merged image information (i.e., one-level IDWT operations, the term $LL_{i}\left(x,y\right)$ estimated from Equation (17) with i=0). Similarly, the merged image information and the recovered pixel-based image by the corresponding IDWT decoding process of min-max and mean-mean wavelet image fusion can be seen in Figure 11 and Figure 12, respectively (also see Equations (9)–(17)). Note that, as mentioned before, such IDWT-decoded images generated from max-min, min-max or mean-mean wavelet image fusion (five-level wavelet transform calculations employed in this work (i.e., the IDWT initial setting i=4 set in Equation (17)) will then further be carried out for VGG-16 CNN deep learning and recognition.

## 4. Experiments

Hand gesture intention actions recognition experiments were made in a laboratory office environment. In the phase of gesture action database establishment, the specific subject was requested to record the indicated hand gesture intention actions. The subject enlisted for hand gesture database establishment was male and about 20 years of age. Table 1 shows the indicated ten continuous-time hand gesture action categorizations, each of which represents the specific semantic human intention behavior in the real life of laboratory environments. Note that each of these gesture actions operated is well-designed according to the standard sign language (specific sign language used in social actions of Taiwan) definition in [27]. These actions of Action-1, Action-2, …, and Action-10 are essentially common in an interaction scenario of a laboratory office composed of student members and the leading teacher, and very beneficial for the smart social activities of a normal group or smart person communication between the normal and the disabled, which are “To the restroom!,” “What?”, “Good bye!”, “Is it ok?”, “Good morning!”, “The teacher!”, “The exam!”, “Hello!”, “Calm down!”, and “Exchange!”, respectively. Examples of the ten included designed hand gesture intention actions in a laboratory office interaction are listed as follows (smart interactions between the normal using acoustic voices and the disabled using hand gesture actions):
(The normal): Where are you going?(The disabled): To the restroom!(The normal): Do you remember?(The disabled): What?(The normal): I’m going back first.(The disabled): Good bye!(The normal): Ok, finished!(The disabled): Is it ok?(The normal): Hi, good morning!(The disabled): Good morning!(The normal): Who is him?(The disabled): The teacher!(The normal): Is it an exam or quiz?(The disabled): The exam!(The normal): Hello!(The disabled): Hello!(The normal): I’m so excited now.(The disabled): Calm down!(The normal): Is academic exchange required?(The disabled): Exchange!

**Figure 13:**
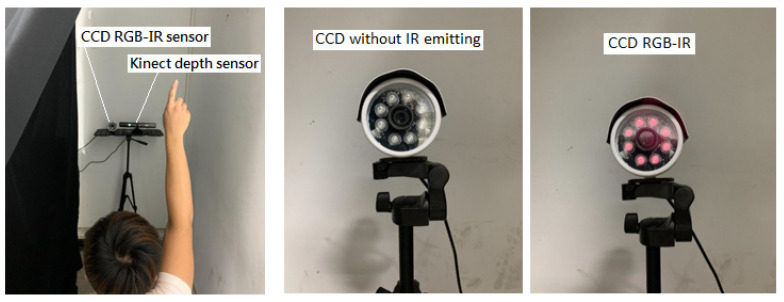
Acquisitions of the hand gesture intention images of different modalities of RGB-IR and depth-grayscale from a CCD camera and a Kinect device.

Note that these captured hand gesture actions in Table 1 belong to the type of CCD RGB-IR, captured using a CCD camera in a situation of almost complete darkness. Figure 13 reveals that the specific subject performs the indicated gesture action in a proper location to the deployed image sensor. Note that, in Figure 13, when the CCD camera operates in dark conditions, i.e., in insufficient light, the IR lights around the image lens of the CCD will keep emitting to ensure visible rendering of the RGB images (see the right of Figure 13). In total, 60,000 action images were collected, mainly including two separated parts: 30,000 action images of CCD RGB-IR and 30,000 action images of Kinect depth-grayscale; the collected subject was to make an action for each of the indicated 10 types of hand gesture intention actions, with each specific type of action performed 50 times (25 times for actions for deep learning model training and the other 25 times actions for the deep learning model test). Each gesture action is a completed image capture in the time period of 2 s with a frame rate of 30. The established database is used for action recognition performance evaluations of the presented VGG-16 CNN deep learning incorporated with wavelet image fusion of CCD RGB-IR and depth-grayscale modalities.

As mentioned, this work employs 5-level wavelet image fusion for hybridizing two different sensor modality data. Before VGG-16 CNN deep learning and recognition, each of the 30,000 CCD RGB-IR action images collected was made into a wavelet image fusion of “max-min”, “min-max”, and “mean-mean” with the corresponding image of the same time-stamp in another collected set of 30,000 depth-grayscale action images. In total, there are 30,000 “max-min” wavelet fused images, 30,000 “min-max” wavelet fused images and 30,000 “mean-mean” wavelet fused images for making the recognition and testing the three different VGG-16 CNN deep learning models (for each VGG-16 CNN, 15,000 action images used for model training and the other 15,000 action images used for model test). In the phase of VGG-16 CNN model training, the related hyper-parameter settings were finely made, the batch size set to 50, the learning rate set to 0.0001, the training ratio set to 0.8 and the number of epochs set to 60; in addition, for minimizing the value of the training loss, a popular optimizer, “Nesterov Momentum”, with the value of momentum set to 0.9, was also adopted in training of VGG-16 CNN. The specifications of the related hardware devices employed in the developed hand gesture intention recognition system are as follows: a desktop PC with a Windows 10 OS, equipped with an i7-8700 3.20 GHz CPU (Intel, Santa Clara, CA, USA), 16 GB RAM and a graphics card with a Geforce GTX 1080 Ti GPU (Nvidia, Santa Clara, CA, USA); a CCD camera for capturing the CCD RGB-IR hand gesture images was connected to a monitor host with four surveillance channels (only one channel used in this work). In total, 8 IR-LEDs were deployed around the central image sensor in the CCD camera. The AVI video recorded from the CCD camera has the specific format of H.265, 1920 × 1080 and 255 kbps. Note that the AVI video then performed extractions of H.265-encoded images and decoded images (RGB-IR images with specific time-stamps finally obtained for recognition system developments). The Microsoft Kinect sensor device for acquisitions of depth-grayscale images belonged to the Kinect v1 type, where the effective range for depth capturing is from 0.5 m to 4.5 m. In this work of acquiring the depth-grayscale gesture images, the distance between the gesture-making subject and the sensor device is about 1.25–1.30 m. The Kinect for Windows software development kit (SDK) v1.8 was used in this work.

**Figure 14:**
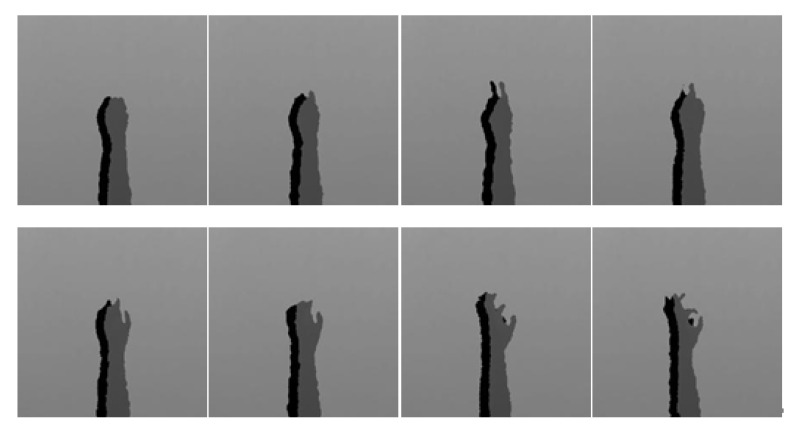
The sensor modality of “depth-grayscale” of the hand gesture intention action, Action 1.

**Figure 15:**
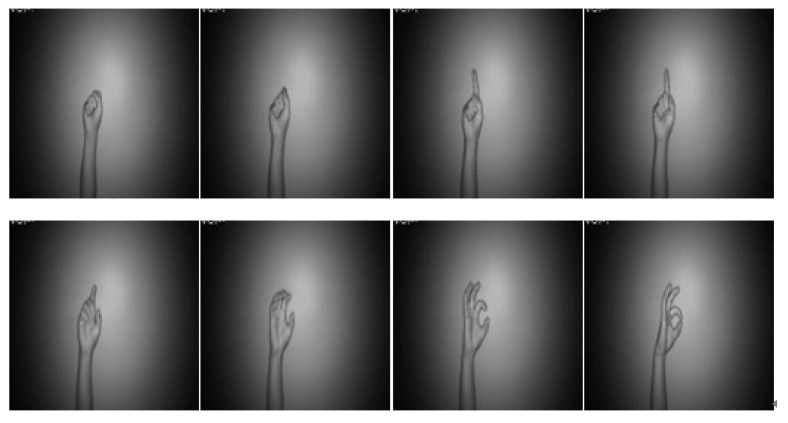
The sensor modality of “CCD RGB-IR” of the hand gesture intention action, Action 1.

**Figure 16:**
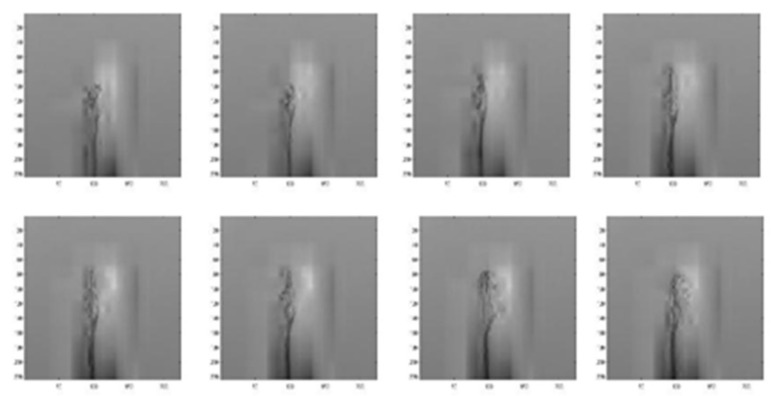
The fused image of two different sensor modalities of “Depth-Grayscale” and “CCD RGB-IR” by wavelet image fusion with max-min operations for VGG-16 CNN deep learning and recognition (e.g., the hand gesture intention action, Action 1).

**Figure 17:**
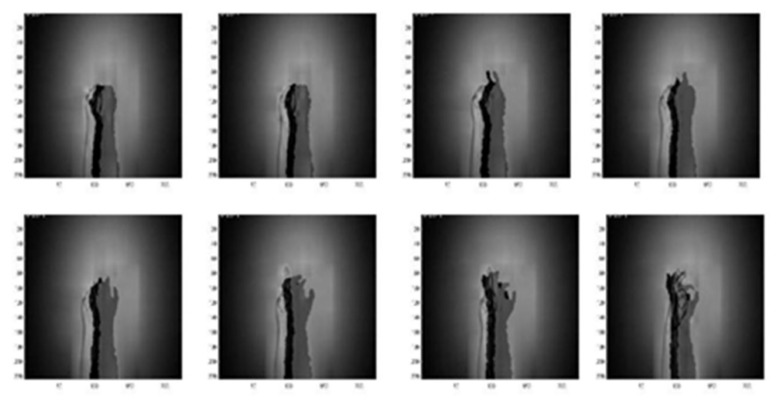
The fused image of two different sensor modalities of “Depth-Grayscale” and “CCD RGB-IR” by wavelet image fusion with min-max operations for VGG-16 CNN deep learning and recognition (e.g., the hand gesture intention action, Action 1).

**Figure 18:**
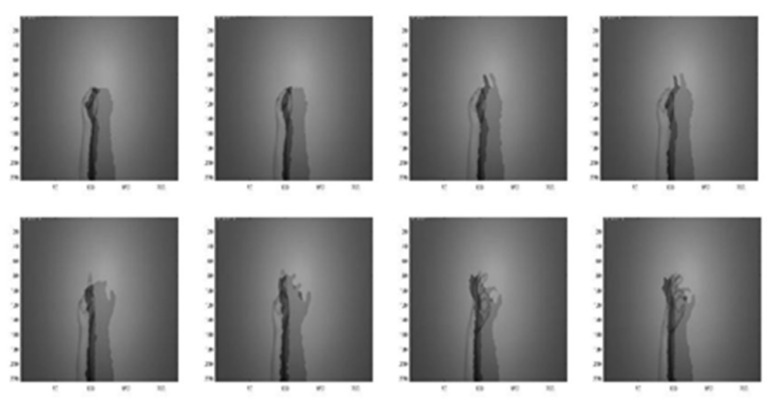
The fused image of two different sensor modalities of “Depth-Grayscale” and “CCD RGB-IR” by wavelet image fusion with mean-mean operations for VGG-16 CNN deep learning and recognition (e.g., the hand gesture intention action, Action 1).

Figure 14, Figure 15, Figure 16, Figure 17 and Figure 18 show an example of the 5-level wavelet image fusion of the two different sensor modalities of hand gesture action images used in this study. For simplicity, only wavelet transform fused images of the “Action 1” type of hand gesture intention actions are provided. Figure 14 and Figure 15 are the original single modality of the image data captured from the depth sensor and the CCD camera, respectively. The merged images of “max-min”, “min-max” and “mean-mean” wavelet image fusion of the “Action 1” CCD RGB-IR and depth-grayscale images are given in Figure 16, Figure 17 and Figure 18, respectively. It is also noted that the wavelet fusion merged images are then used for training and recognition of the CNN deep learning.

**Figure 19:**
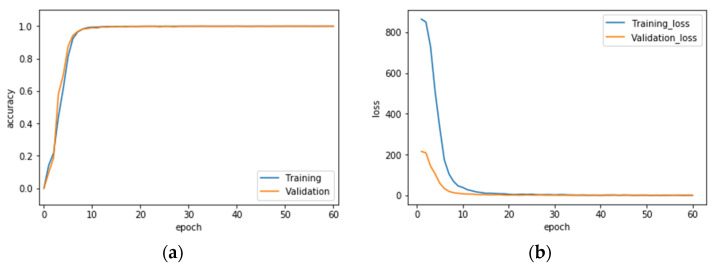
Recognition rate (**a**) and loss value (**b**) curves of the VGG-16 CNN model training using max-min wavelet fused images.

**Figure 20:**
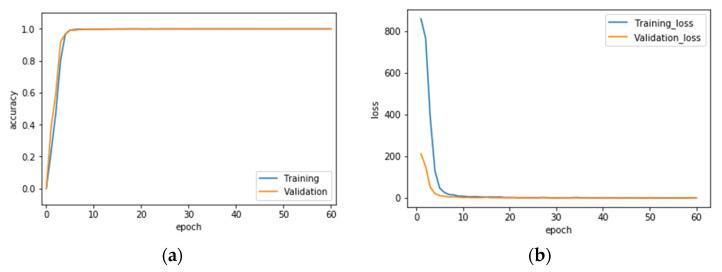
Recognition rate (**a**) and loss value (**b**) curves of the VGG-16 CNN model training using min-max wavelet fused images.

**Figure 21:**
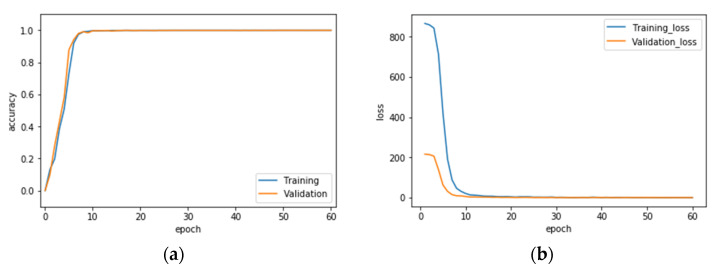
Recognition rate (**a**) and loss value (**b**) curves of the VGG-16 CNN model training using mean-mean wavelet fused images.

Finally, the VGG-16 CNN recognition performance results of hand gesture intention recognition by the conventional single modality and the presented hybridized modality by wavelet image fusion are presented. Table 2 provides the averaged recognition performance of the overall ten classes of gesture actions of all hand gesture intention recognition strategies with (or without) image fusion in the phase of VGG-16 CNN training. It can be clearly seen that each strategy achieves complete recognition of 100%. Table 3 is the recognition performance of hand gesture intention recognition by the traditional VGG-16 CNN with only one modality of CCD RGB-IR or depth-grayscale images; the recognition effectiveness of the presented VGG-16 CNN incorporated with wavelet image fusion of CCD RGB-IR and depth-grayscale on hand gesture intention recognition is given in Table 4. Observations of the recognition performance outcomes, revealed by Table 3 and Table 4, can be made from two different viewpoints: a separate, one class of action, and the overall ten classes of actions. From the viewpoint of a separate, one class of action, two classes of gesture actions, Action 1 and Action 10, can be improved the recognition performance of VGG-16 CNN with only the single modality CCD RGB-IR and depth-grayscale using either of max-min, min-max and mean-mean wavelet image fusion strategies on CCD RGB-IR and depth-grayscale. In addition, two hybridization strategies, VGG-16 CNN with min-max and mean-mean wavelet image fusion, can significantly improve six classes of actions, which are Actions 1, 4, 5, 6, 9 and 10 and Actions 1, 3, 4, 6, 9 and 10, respectively. For VGG-16 CNN with max-min wavelet image fusion, the number of gesture classes that can increase the recognition accuracy is 3 (Actions 1, 5 and 10). Observed from Table 3 and Table 4, it is also noted that both Actions 3 and 5 still have substandard recognition rates, even with the use of wavelet image fusion on CCD RGB-IR and depth-grayscale. Such a phenomenon of difficulty in performance improvement is extremely reasonable. Without considerations of image fusion, Action 3 with the modality of CCD RGB-IR and depth-grayscale only has a performance of 40.33% and 17.93%, respectively. For Action 5, recognition performances of the single modality CCD RGB-IR and depth-grayscale are much worse, just 2.67% and 25.20%, respectively. That both types of single modality actions are hard to be categorized or even completely unrecognizable will not benefit from the data complementary effect of wavelet image fusion. Regarding the overall consideration of all ten classes of actions, VGG-16 CNN recognition incorporated with wavelet image fusion of min-max and mean-mean merged strategies have apparently more outstanding recognition performances, both of which are significantly higher than those of CCD RGB-IR alone and depth-grayscale alone. VGG-16 CNN with min-max wavelet fusion performs best, achieving 83.88%, followed by 80.95% for the mean-mean wavelet fusion. However, VGG-16 CNN with max-min wavelet image fusion has no apparent effects on improvements of averaged recognition accuracy, reaching only 73.91%. The averaged recognition rate of VGG-16 CNN using only a single-sensor modality without any image fusion is not satisfactory—75.33% with CCD RGB-IR recognition and 72.94% with depth-grayscale recognition. Experimental results clearly reveal the effectiveness of the presented deep learning using wavelet image fusion with proper merge strategies of the wavelet decomposition parameter sets for improvements of the averaged recognition accuracy of hand gesture intention action classifications. In addition, confusion matrices of VGG-16 CNN with max-min, min-max and mean-mean wavelet image fusion are also provided in this work, which can be seen in Table 5, Table 6 and Table 7, respectively. Figure 19, Figure 20 and Figure 21 show the recognition rate and loss value curves of VGG-16 CNN model training (as mentioned, totally 60 iterations set in the training phase) using max-min, min-max and mean-mean wavelet fused images, respectively. For demonstrating the competitiveness of the presented approach on computation speed, the time information of both the training and test phases of the hand gesture intention recognition using the proposed VGG-16 CNN with wavelet image fusion is also provided in Table 8. It is clearly seen in Table 8 that for each of VGG-16 CNN with max-min, min-max and mean-mean wavelet image fusion, the averaged time of a gesture intention action required for recognition calculations is about a half-second, which can achieve real-time computation in real-life applications.

## 5. Discussions

In this work, as mentioned, wavelet image fusion for hybridizing both CCD RGB-IR and depth-scale images belongs to the DWT-based approach. Integer wavelet transform (IWT) will perhaps be also an alternative for extracting wavelet decomposition coefficients. Although IWT has some competitive properties, such as fast computation and efficient memory usages, the IWT technique is seen to have more applications in multimedia fields of data compression with lossless recovery and data hiding with high security. Recently, IWT has also been further integrated with DWT for image fusion in the application of image steganography constructions with data concealments in multimedia communication [28]. The feasibility of IWT used in the field of pattern recognition with multimodality fusion and deep learning may be evaluated in future work.

The deep learning model for recognition of hand gesture intention actions adopted in this work is the VGG-16 CNN due to the wide usage of VGGNet-type deep learning models in the biometric image-based pattern recognition field (e.g., face recognition, fingerprint recognition, iris recognition and hand gesture recognition in this work). In fact, some possible extended works may be further explored to use other types of CNN structures for evaluating the performances.

In the study, as mentioned, compared with the single modality of RGB-IR or depth-scale data for VGG-16 CNN deep learning and recognition, the presented VGG-16 CNN with wavelet image fusion may significantly increase the averaged recognition accuracy in proper DWT decomposition merge calculations. The main patterns to be classified by the recognition system are hand gesture intention actions in only a very short time-period (simple gesture actions that are extremely less time consuming, as mentioned in Table 1). In the situation of continuous-time dynamic hand gesture actions with long operation time-periods and many sensitivities given context dependency (generally called as sign language actions), for recognition, a dynamic deep learning model, such as the well-known LSTM or the more advanced structure of CNN-LSTM, belonging to the recursive neural network (RNN) type [29], will be required for maintaining satisfactory recognition accuracy. The future work of this study will consider the development of sign language recognition systems where such dynamic deep learning structures will be further explored and evaluated.

Finally, the technical issue that the presented system will be used in a real-world application is discussed. As mentioned, the gesture-making operator is requested to complete each indicated gesture action in a fixed time of 2 s in this work. However, from the view-point of practical applications in real life, it will not make sense. As popular voice control-based speech recognition seen in device control applications nowadays, a waking-up and terminating scheme for the gesture recognition system will be inevitably required to be able to accurately extract the significant hand gesture intention action made by the subject. An earlier study of the author on the establishment of an expert system for the sport instructor robot with Kinect sensor-based gesture recognition has investigated gesture activity detection (GAD) [2] where various effective GAD methods for extractions of significant gesture actions are presented. Future work will explore this issue for further promoting developed hand gesture intention to be practically used. On the other hand, in practical applications, the promoted system will also additionally take into consideration designs and integrations of the anti-model of hand gesture intention actions, to tolerate the occurrence of unexpected gesture actions (e.g., an action that is out of the pre-defined database of 10 actions in this work) made by the subject.

## 6. Conclusions

In this study, a deep learning framework, VGG-16 CNN, incorporated with a 5-level wavelet image fusion of CCD RGB-IR and depth-grayscale sensor data streams, is proposed for hand gesture intention recognition. Experimental results demonstrate that the hybridization of CCD RGB-IR and depth-grayscale information by the min-max data fusion strategy to merge wavelet decomposition performs best and is significantly superior to only CCD RGB-IR without any depth-grayscale data fused on the recognition accuracy of VGG-16 CNN deep learning. The presented approach can achieve competitive performances in a surveillance application with human gesture intention recognition. Finally, for the possible extension of this work in the future, the presented approach of CNN deep learning with wavelet image fusion in this study can be further enhanced by incorporating additional modalities of sensing information (such as the well-known IMU or SEMG data from wearable watches or bracelets) to promote the system to be able to perform sign language recognition with semantic context dependency or more complex human activity behavior recognition with social actions of multiple subject interactions.

## Figures and Tables

**Table 1:** A database of ten continuous-time hand gesture intention actions established for evaluation of the presented deep learning with wavelet-based image fusion (the modality of CCD RGB-IR) [27].

Action Classes	Data Streams of Continuous-Time CCD RGB-IR Hand Gesture Actions
Action 1 (To the restroom!)	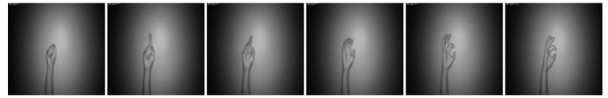
Action 2 (What?)	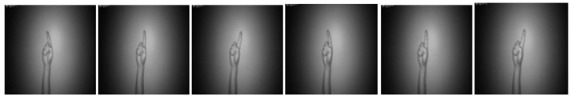
Action 3 (Good bye!)	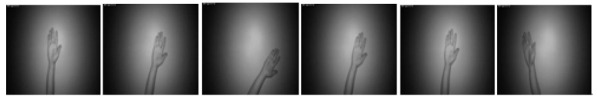
Action 4 (Is it ok?)	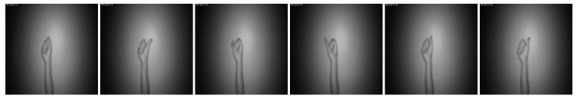
Action 5 (Good morning!)	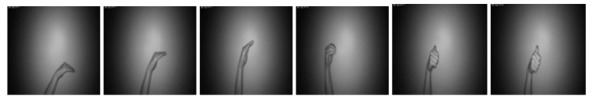
Action 6 (The teacher!)	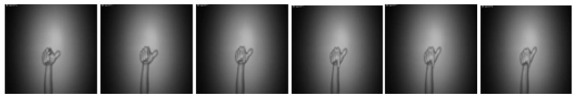
Action 7 (The exam!)	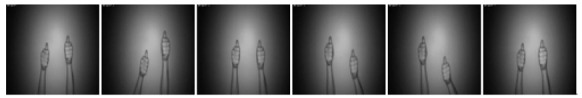
Action 8 (Hello!)	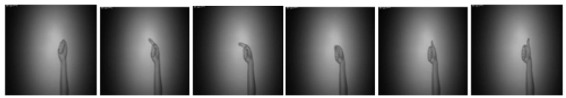
Action 9 (Calm down!)	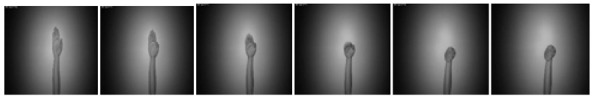
Action 10 (Exchange!)	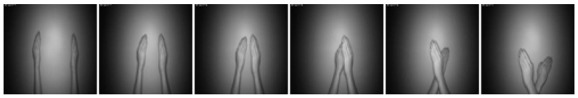

**Table 2:** Recognition performances of hand gesture intention recognition by typical deep learning of a VGG-16 CNN with only a single modality of CCD RGB-IR or depth-grayscale images and the proposed VGG-16 CNN deep learning with wavelet image fusion of CCD RGB-IR and depth-grayscale by “max-min”, “min-max” or “mean-mean” merge types on recognition system training.

Data Modality	CCD RGB-IR	Depth-Grayscale	Wavelet Fusion Using Max-Min	Wavelet Fusion Using Min-Max	Wavelet Fusion Using Mean-Mean
Average	100%	100%	100%	100%	100%

**Table 3:** Recognition performances of hand gesture intention recognition by typical deep learning of the VGG-16 CNN with only a single modality of CCD RGB-IR or depth-grayscale images on the recognition system test.

Single Sensor Modality	CCD RGB-IR	Depth-Grayscale
Action-1	67.67%	93.33%
Action-2	100.00%	99.93%
Action-3	40.33%	17.93%
Action-4	59.67%	69.60%
Action-5	2.67%	25.20%
Action-6	86.33%	95.26%
Action-7	100.00%	77.66%
Action-8	100.00%	91.46%
Action-9	100.00%	98.00%
Action-10	96.67%	61.06%
Average	75.33%	72.94%

**Table 4:** Recognition performances of the hand gesture intention recognition using the presented dual-channel VGG-16 CNN with wavelet-based image fusion of two different modalities of data, CCD RGB-IR and depth-grayscale, on the recognition system test (by three different merged types of wavelet decompositions).

Action Categorization	Wavelet Fusion of the Max-Min Type	Wavelet Fusion of the Min-Max Type	Wavelet Fusion of the Mean-Mean Type
Action-1	98.20%	99.53%	99.20%
Action-2	94.27%	99.07%	97.87%
Action-3	6.00%	24.20%	44.87%
Action-4	43.93%	84.80%	71.13%
Action-5	44.33%	37.53%	18.13%
Action-6	72.33%	98.33%	99.27%
Action-7	82.13%	99.00%	81.67%
Action-8	98.40%	98.33%	99.13%
Action-9	99.47%	100.00%	100.00%
Action-10	100.00%	98.00%	98.20%
Average	73.91%	83.88%	80.95%

**Table 5:** The confusion matrix of hand gesture intention recognition by VGG-16 CNN deep learning with wavelet image fusion of the merge type of “max-min” on the recognition system test.

Max-Min	1	2	3	4	5	6	7	8	9	10
1	1473	0	0	11	16	0	0	0	0	0
2	0	1414	1	0	0	0	0	84	1	0
3	132	0	90	7	1003	29	239	0	0	0
4	41	0	25	659	47	456	10	0	0	262
5	107	0	13	0	665	0	665	0	0	50
6	4	302	18	0	6	1085	24	44	5	12
7	0	0	0	0	0	241	1232	2	10	15
8	0	24	0	0	0	0	0	1476	0	0
9	0	0	0	8	0	0	0	0	1492	0
10	0	0	0	0	0	0	0	0	0	1500

**Table 6:** The confusion matrix of hand gesture intention recognition by VGG-16 CNN deep learning with wavelet image fusion of the merge type of “min-max” on the recognition system test.

Min-Max	1	2	3	4	5	6	7	8	9	10
1	1493	0	0	7	0	0	0	0	0	0
2	0	1486	0	0	0	0	0	13	1	0
3	161	0	363	637	322	17	0	0	0	0
4	26	2	35	1272	7	75	1	0	2	80
5	0	0	192	0	563	0	292	0	0	453
6	0	0	4	0	8	1475	13	0	0	0
7	0	0	0	6	0	0	1485	0	0	9
8	0	25	0	0	0	0	0	1475	0	0
9	0	0	0	0	0	0	0	0	1500	0
10	12	0	0	18	0	0	0	0	0	1470

**Table 7:** The confusion matrix of hand gesture intention recognition by VGG-16 CNN deep learning with wavelet image fusion of the merge type of “mean-mean” on the recognition system test.

Mean-Mean	1	2	3	4	5	6	7	8	9	10
1	1488	0	0	12	0	0	0	0	0	0
2	0	1468	0	0	0	0	0	32	0	0
3	506	0	673	131	74	50	66	0	0	0
4	25	1	107	1067	0	252	14	0	34	0
5	5	0	712	0	272	0	192	0	0	319
6	3	0	7	0	0	1489	1	0	0	0
7	0	0	0	27	0	58	1225	96	0	94
8	0	13	0	0	0	0	0	1487	0	0
9	0	0	0	0	0	0	0	0	1500	0
10	16	0	0	11	0	0	0	0	0	1473

**Table 8:** Training and test time of the hand gesture intention recognition of the proposed VGG-16 CNN deep learning with wavelet image fusion of CCD RGB-IR and depth-grayscale using “max-min”, “min-max” or “mean-mean” merge types on the hand gesture database (10 classes of gesture intention actions with 25 actions collected in each class contained in each of the training and test databases).

Merge Types of Wavelet Image Fusion for VGG-16 CNN	Wavelet Fusion Using Max-Min	Wavelet Fusion Using Min-Max	Wavelet Fusion Using Mean-Mean
Training time in total	9067.80 s	9070.75 s	9096.67 s
Test time in total	133.96 s	128.17 s	128.57 s
Test time (averaged, one-action)	0.54 s	0.51 s	0.51 s

## Data Availability

Not applicable.
